# Recurring Luxation of the Lower Jaw

**Published:** 1891-04

**Authors:** 


### Recurring Luxation of the Lower Jaw.

At the Academy of Medicine, New York, they have discovered that luxation of the jaw may occur from more than one cause. At a meeting of the section on surgery, "Dr. W. R. Townsend presented a woman fifty-three years of age, who had been much troubled by recurring dislocation of the lower jaw since about five years ago, when a dentist used some force in taking a cast of the upper jaw. She could now keep the jaws in normal apposition only by holding the mouth shut, and during sleep the lower jaw would slip forward." (*Medical Record*).

Force in taking an upper impression causing dislocation of the lower jaw, *nonsense!* Any student of anatomy knows that any amount of force upon the upper jaw can not dislocate it from the lower. We have heard of dislocation occurring from taking an impression of the upper jaw but it was not caused by unnecessary force, it occurred the same way that another woman's jaw was dislocated, by simply gapping, *i. e.* opening the mouth too wide.

We fail to see where the force would come in in taking an upper impressison. W.

---

The diplomas granted by the Royal College of Surgeons of Dublin are: Fellow and licentiate, also licentiate in dental surgery. The former two are full surgical diplomas and entitle their possessor to practice in Great Britain, while the latter is the regular dental qualification which, with two similar ones in Scotland and one in Canada, constitute the only dental qualifications granted in the kingdom. Harvard and Michigan dental degrees are the only registrable diplomas in dentistry.

One of Ann Arbor's graduates has been appointed to a professorship in Heidleburg University. This is the first instance of such an honor being bestowed on an American student.

The above from a Chicago paper is not in exact accordance with the facts. It is pretty well understood, all round in this vicinity, that Dr. W. D. Miller, who is also a graduate of the

University of Michigan, is and has been for several years a professor in the University of Berlin; and in addition to this the world knows that Dr. Miller has done something since he has occupied that position that commands universal admiration, and places him as an investigator before the scientific world without an equal in his special line.

ON the 10th of March the building occupied by the Buffalo Dental Manufacturing Company was destroyed by fire. The large number of standard and useful articles supplied to the profession by this old reliable concern, entitle it to the sincere sympathy of every dentist whose labor has been made easier or more efficient, because of the ingenuity and enterprise it has always manifested. We are pleased to learn that the old Company has been re-organized and will continue the business with new and improved facilities; and the continuation of Dr. Theo. G. Lewis and Col. John E. Robie, with the new organization will assure the profession that no backward step is contemplated.

THE *Dental Review's* New York correspondent objects to editor Hungerford's criticism of his "unclosing laudation" of certain prominent members of the profession in the east. We don't object to his laudation of any one in particular or in general either, but we would enjoy his letters more if his subject matter was more systematically treated, and his personal and general allusions were not so localized as to be destitute of sense or interest to us western folks. There are several exceedingly interesting matters though in his letter in the March *Review*, for which we are duely grateful, so much so, that we think it desirable to give them in condensed form to our readers.

DR. MAIN the 5th Avenue dentist says he has had an average annual income of $20,000 from his practice for twenty years, why can't he help Bodecker build the Academy of Dentistry?

Our correspondent closes with an exhortation to young men to take their places in the work of dental society, dental journalism &c, and not defer this duty from fear of adverse criticism, which is often only commendation with desire that the strength of the young man's mental ability may be developed. We hope our venerable correspondent (for we find our friend was practicing dentistry before we were born and contributed an article to the *Cosmos* when we were only four years old, although the first number of the *Cosmos* was not printed until a year later) will take kindly our criticism, for we really enjoy his letters and we know the time and effort he may put into them is not wasted, and we are sure editor Harlan will not shut off so interesting a writer.

It is contrary to the code of ethics of the American Dental Association, and consequently of all delegate dental societies, to give a public testimonial or indorsement to a patented or secret nostrum, amalgam or anything that may be used to the commercial advantage of its owner; thus rendering the practice of giving testimonials unprofessional and disreputable. How far then must the moral sentiment be bent to allow the editor to accept a free copy of a book, an instrument, or any commercial commodity in exchange for a favorable review or comment which will be used to encourage traffic and financially benefit the owner? Does it not need considerable assurance, for the publisher of a book or even a professional journal, to ask gratuitously an indorsement of which is to be used as an advertisement? Such things are done.

Dr. W. C. Barret has procured and sold to the New York Odontological Society, a complete collection of skulls, representing all mammalia and fishes having teeth. Truly a valuable and instructive collection, such as every dental school should possess. For the present this collection will be housed in the new and magnificent building of the New York Academy of Medicine, where it will probably remain until the Bodecker Academy of Dentistry shall have been built.

Speaking of Assurance, does it not seem as though it was a little too much to have the editor of a professional journal soliciting practitioners, scientific workers, teachers, editors, &c., to give within the limits of a postal card, methods of treatment, or opinions on subjects that are already exhaustively treated in text books or professional literature? It takes time and brains to condense into the limits of a postal card, what would require several pages to give an intelligible and helpful answer to some of these questions, and we don't think even an editor has any business to ask such favors even for the good of the profession, without due compensation. It seems to us a better way would be to employ a capable man to search out replies to these questions from articles contributed to the journals and from reliable text books, quoting and giving due credit. This would accomplish the purpose without imposing upon the good nature of busy people.

One More, a certain extensively circulated dental journal has a habit of copying an article from a journal published twenty to thirty years ago, giving due credit to the author who always is one of our distinguished men, but giving no credit to the journal from which the article is taken or the date; thus doing an injustice to the author whose views of the subject may have changed, because of more recent investigations in science or practice; and imposing upon the younger members of the profession the antiquated ideas and views of men whose thoughts they are accustomed to consider valuable, and worthy of imitation. This isn't enterprise or journalism, it is a bad practice and should not be tolerated.

The *Dental Review* is authority for the statement that a man named Emanuel, and a lawyer by profession, living at Aiken, South Carolina, has discovered a process by which he can separate aluminum from the native clay of his country at an expense of only two dollars and fifty cents per ton. If this thing keeps on some one will have to turn his attention toward the problem of preventing the transformation of the earth into aluminum. H.

THE NEW YORK DENTAL COLLEGE is to have a new home, having purchased one for $106,000. We are truly glad of this, for the old building must have been totally incapable of properly housing and instructing the large classes that have gathered within its walls during the last four or five years.

DR. ASHLEY FAUGHT has made an ingenious device for heating and packing gutta-percha without injuring it by overheating. Dr. C. M. Richmond has invented an apparatus for obtunding purposes, involving the principle of the application of medicated steam spray to the part to be obtunded.

